# P03 An evaluation of Antibiotic Champions events in GP practices in Cambridgeshire and Peterborough Integrated Care System

**DOI:** 10.1093/jacamr/dlae136.007

**Published:** 2024-08-23

**Authors:** J Patel, S Ransom, C Bennet, S Culloty, C Micallef

**Affiliations:** Cambridgeshire and Peterborough Integrated Care Board, Ely, Cambridgeshire, UK; Cambridgeshire and Peterborough Integrated Care Board, Ely, Cambridgeshire, UK; Cambridgeshire and Peterborough Integrated Care Board, Ely, Cambridgeshire, UK; Cornford Surgery, Cherry Hinton Road, Cambridge, UK; Cambridgeshire and Peterborough Integrated Care Board, Ely, Cambridgeshire, UK; Jenner Healthcare, The Medical Centre, Peterborough, UK; Cambridgeshire and Peterborough Integrated Care Board, Ely, Cambridgeshire, UK

## Abstract

**Background:**

In 2022, all GP practices in the area were asked to nominate an Antibiotic Champion for each practice. These Antibiotic Champions were then asked to write actions plans for their antimicrobial stewardship (AMS) initiatives and invited to quarterly virtual educational meetings: Antibiotic Champion events. The first event was launched on 2 November 2022.

**Objectives:**

We sought to understand whether Antibiotic Champions attending the Antibiotic Champions events were finding these events useful to their practice and whether they wanted additional support to deliver their antibiotic prescribing targets.

**Methods:**

Following the 8 November 2023 Antibiotic Champion event, we disseminated electronic evaluation forms, using MS Forms to all attendees via the Training Hub which arranges the logistics for the virtual meetings. This e-survey was available from 8 February to 3 December 2023 and regular reminders were sent by the Training Hub.

**Results:**

From 68 participants, 21 responded (30.1% response rate). A total of 66.7% (14/21) identified as medical doctors with 28.6% (6/21) declaring that they were pharmacists and 5% (1/21) advanced clinical practitioners. There was no representation from the nursing or pharmacy technician professions. In all, 90.5% (19/21) declared that they were prescribers and 76% (16/21) prescribed antimicrobials in their roles. The majority of respondents—71.4% (15/21)—had been in the NHS for >10 years. Figure 1 portrays the feedback from respondents on further support needed to deliver AMS metrics whilst Figure 2 delineates respondent satisfaction for the Antibiotic Champion event meetings. Finally, Figure 3 outlines AMS activities in GP practices. In November 2022, the national primary care metrics for total antibiotic prescribing (0.871) and broad-spectrum antibiotic (10%) were not being achieved locally but national ePACT data issued in November 2023 highlighted that the broad-spectrum metric was now being achieved (9.92%).

**Conclusions:**

This study presents an easy, cost-effective way of engaging with healthcare professionals in GP practices, over a large and varied geographical area, on antimicrobial stewardship initiatives. We suggest that these events may have helped support GP practices to deliver their AMS targets.

**Figure 1.**
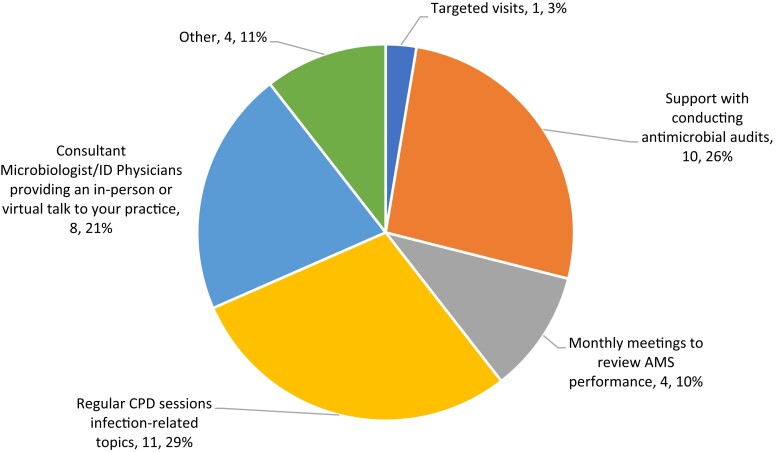
Feedback from respondents on further support needed to deliver AMS metrics.

**Figure 2.**
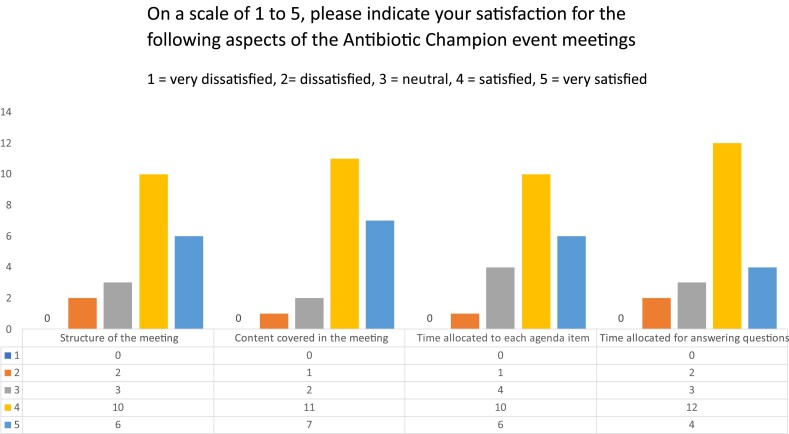
Respondent satisfaction for the Antibiotic Champion Event meetings.

**Figure 3:**
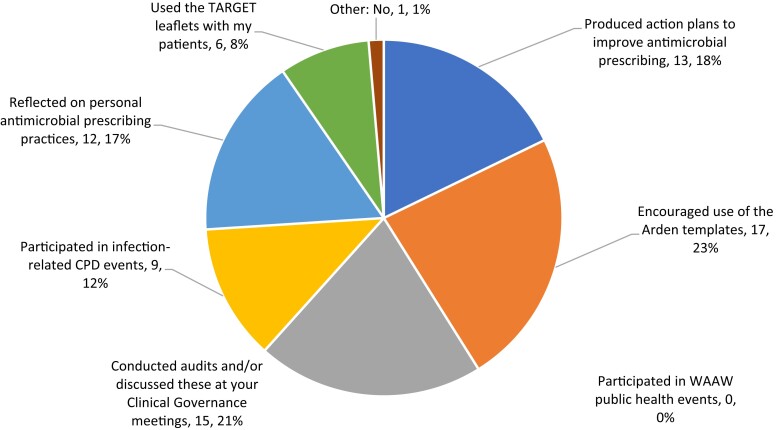
AMS Activities in GP practices.

